# Relative humidity and qualities of hygroscopic medications stored in different one-dose packaging papers

**DOI:** 10.1016/j.heliyon.2020.e05362

**Published:** 2020-11-06

**Authors:** Taisuke Matsuo, Yusei Yoshida, Takashi Tomita, Yasuyuki Sadzuka

**Affiliations:** a Division of Advanced Pharmaceutics, Department of Clinical Pharmaceutical Sciences, School of Pharmacy, Iwate Medical University, 1-1-1, Idaidori, Yahaba-cho, Shiwa-gun, Iwate, 028-3694, Japan; b Department of Pharmaceutical Sciences, Faculty of Pharmaceutical Sciences, Teikyo Heisei University, Japan

**Keywords:** One-dose package, Cellophane polyethylene laminating paper, Glassine paper, Moisture absorption, Relative humidity, Pharmaceutical science, Public health, Clinical research, Health promotion, Social sciences

## Abstract

The one-dose package is useful for patients who are prescribed multiple medications. However, the one-dose packaging of hygroscopic medications is difficult because the quality of the medication is reduced by moisture absorption. Cellophane polyethylene laminating paper at 20 μm or 30 μm thickness and glassine papers are widely used for one-dose packaging. The basic characteristics, such as water permeability, of these packaging papers have been demonstrated by companies; however, the quality changes of hygroscopic medications stored in these packaging papers are poorly understood. In this study, we compared the relative humidity in packaging papers and the qualities of the stored hygroscopic medications among 20 μm and 30 μm thick cellophane polyethylene laminating paper and glassine paper. Glucobay® 50 mg, Magmitt® 330 mg, and Phosblock® 250 mg tablets were used as hygroscopic medications to be packaged and the relative humidity, weight change, and hardness of tablets were measured. The relative humidity decreased in the order of glassine paper, 20 μm thick cellophane polyethylene laminating paper, and 30 μm thick cellophane polyethylene laminating paper. Additionally, tablets inside the 30 μm thick cellophane polyethylene laminating paper gained the least weight. Therefore, tablets in a 30 μm thick polyethylene laminating paper absorb less moisture than those in other papers. However, the effect was less pronounced at high temperature, even if the relative humidity remained the same. We expect that the results will be used by hospitals and clinical pharmacies to understand the characteristics of packaging papers and ensure appropriate usage.

## 1 Introduction

The one-dose packaging of medications improves medication adherence in patients as it prevents forgetting and enables easy administration [1]. It is useful for elderly patients who are prescribed multiple medications [2, 3]. However, the water permeability of cellophane polyethylene laminating paper and glassine paper, which are widely used for one-dose packaging, is higher than that of press through package (PTP) and strip package (SP). The paper used for the one-dose packaging of hygroscopic medications requires attention. Currently, documents such as package inserts, interview forms, and technical books for medications, are consulted to judge whether one-dose packaging is appropriate for a medication. However, most of the information available is based on quality evaluations under unpackaging conditions. In our previous studies, we have demonstrated that the moisture content of hygroscopic medications, such as Glucobay®, Phosblock®, and Magmitt® tablets, stored in 20 μm thick cellophane polyethylene laminating paper is lower than that of non-packaged medications [4, 5]. Additionally, changes in the hardness of tablets and/or appearance of the medications under one-dose packaging conditions are lower than those under non-packaging conditions [4, 5]. Furthermore, the moisture content per tablet in a one-dose package is reduced by the addition of more medications [6]. The type of hygroscopic medication stored in one-dose packages also affects the moisture content per tablet because the hygroscopic property of each medication is different [5, 7]. Thus, it is necessary to evaluate each candidate hygroscopic medication for its suitability for one-dose packaging.

In clinical practice, various packaging papers are used, such as 20 μm and 30 μm thick cellophane polyethylene laminating paper and glassine paper [8, 9]. The basic information, such as moisture permeability, of these papers have been reported by company [9]. However, given that there are differences in the hygroscopic properties of different hygroscopic medication, the relative humidity (RH) associated with different packaging papers and changes in the quality of packaged hygroscopic medications are poorly understood. In this study, we compared the RH and quality changes of hygroscopic medications stored in one-dose packages among 20 μm and 30 μm thick cellophane polyethylene laminating paper and glassine paper. This information will be useful for pharmacists to decide whether hygroscopic medications should be dispensed in a one-dose package or not.

## 2 Materials and methods

### 2.1 Materials

The one-dose packaging papers used in this study were cellophane polyethylene laminating paper at 20 μm (70W cello-poly 20 plane SC-II; Yuyama Co., Ltd., Osaka, Japan; lot: W2 BA9BZ5) and 30 μm (70W cello-poly 30 plane SC-II; Yuyama Co., Ltd.; lot: W3 TA9BZ5) thick and glassine paper (70W glassine plane; Yuyama Co., Ltd.; lot: W WA9CA8). Glucobay® 50 mg (Bayer Yakuhin, Ltd., Osaka, Japan; lot: JPS3558), Magmitt® 330 mg (Kyowa Chemical Industry Co., Ltd., Kagawa, Japan; lot: 19N012), and Phosblock® 250 mg (Kyowa Kirin Co., Ltd., Tokyo, Japan; lot: 19101F) tablets were used as hygroscopic medications. RH was measured using the button-type temperature and humidity data logger “Hygrochron” (KN Laboratories, Inc., Osaka, Japan).

### 2.2 Measurement of RH in one-dose packaging papers without medications

The temperature and humidity data logger was packaged in the three papers. These packaged papers were stored at 25 ± 1 °C and 75 ± 5% RH (Chamber, Constant Temperature and Humidity IG421, Yamato Scientific Co., Ltd., Tokyo, Japan) for 7 days. The RH in each packaged paper was measured at every 3 h (n = 3). The RH in the Constant Temperature and Humidity chamber was also measured without packaging (defined as external environment, n = 2). The results are shown from 3 h to 168 h. Data were not recorded at 0 h to avoid the effects of time taken in measurement setup, packaging of the logger, and setting the Constant Temperature and Humidity chamber.

### 2.3 Qualities of hygroscopic medications in packaging papers stored at 25 °C and 75% RH

The hygroscopic medications packaged in each packaging paper were stored at 25 ± 1 °C and 75 ± 5% RH. The weight and hardness of tablets were measured (n = 5). In consideration of clinical prescriptions, one Glucobay® tablet, two Magmitt® tablets, and four Phosblock® tablets were used per a package and the storage term of Glucobay®, Magmitt®, and Phosblock® tablets was 7, 14, and 14 days, respectively. Hardness of tablet was measured by the Tablet Hardness Tester Monsanto Type (Fujirika Kogyo Co., LTD., Osaka, Japan). The measurement of Glucobay® was conducted on new tablets and on days 1, 3, and 7. In Magmitt® and Phosblock® tablets, the measurements were conducted on new tablets and on days 7 and 14. Additionally, the appearance of Glucobay® tablets was observed after 7 days.

### 2.4 Measurement of RH in one-dose packaging papers with medications stored at 25 °C and 75% RH

The RH in one-dose packages with the medications was measured according to the procedure in Measurement of RH in one-dose packaging papers without medications (n = 3).

### 2.5 Qualities of Phosblock® tablets stored in three types of packaging paper and the RH in these packaging papers at 35 °C and 75% RH

Phosblock® tablets (4 tablets) were packaged in three types of packaging paper, which were stored at 35 ± 1 °C and 75 ± 5% RH. The weight (n = 4) and hardness (n = 5) of the tablets and RH in these papers (n = 3) were measured using the methods described above.

### 2.6 Statistical analysis

The results are shown as mean ± SD. The statistical analyses of weight changes and hardness of tablets were performed by one-way ANOVA with the Tukey–Kramer test using BellCurve for Excel version 3.20 (Social Survey Research Information Co., Ltd., Tokyo, Japan). *P* < 0.05 was a statistically significant difference.

## 3 Results

### 3.1 RH in packaging papers without medications

**Figure 1:**
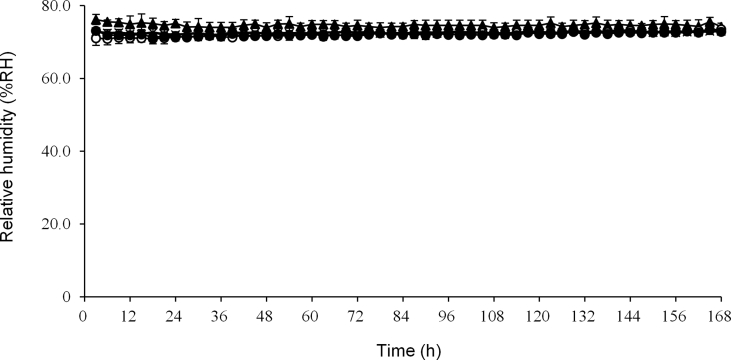
Relative humidity in three packaging papers without medication. The packages were stored under 25 ± 1 °C and 75 ± 5% RH. The relative humidity in packages was measured from 3 h to 168 h. **▲**: external, **〇**: 20 μm thick cellophane polyethylene laminating paper, **●**: 30 μm thick cellophane polyethylene laminating paper, ×: glassine paper. Each point is mean ± SD.

The average RH in 20 μm and 30 μm thick cellophane polyethylene laminating paper and glassine paper without medications from 3 h to 168 h was 72.2%, 72.1%, and 72.6%, respectively. These were similar to the RH of the external environment, which was 74.7% (Figure 1).

### 3.2 Qualities of medications stored in one-dose packages

**Figure 2:**
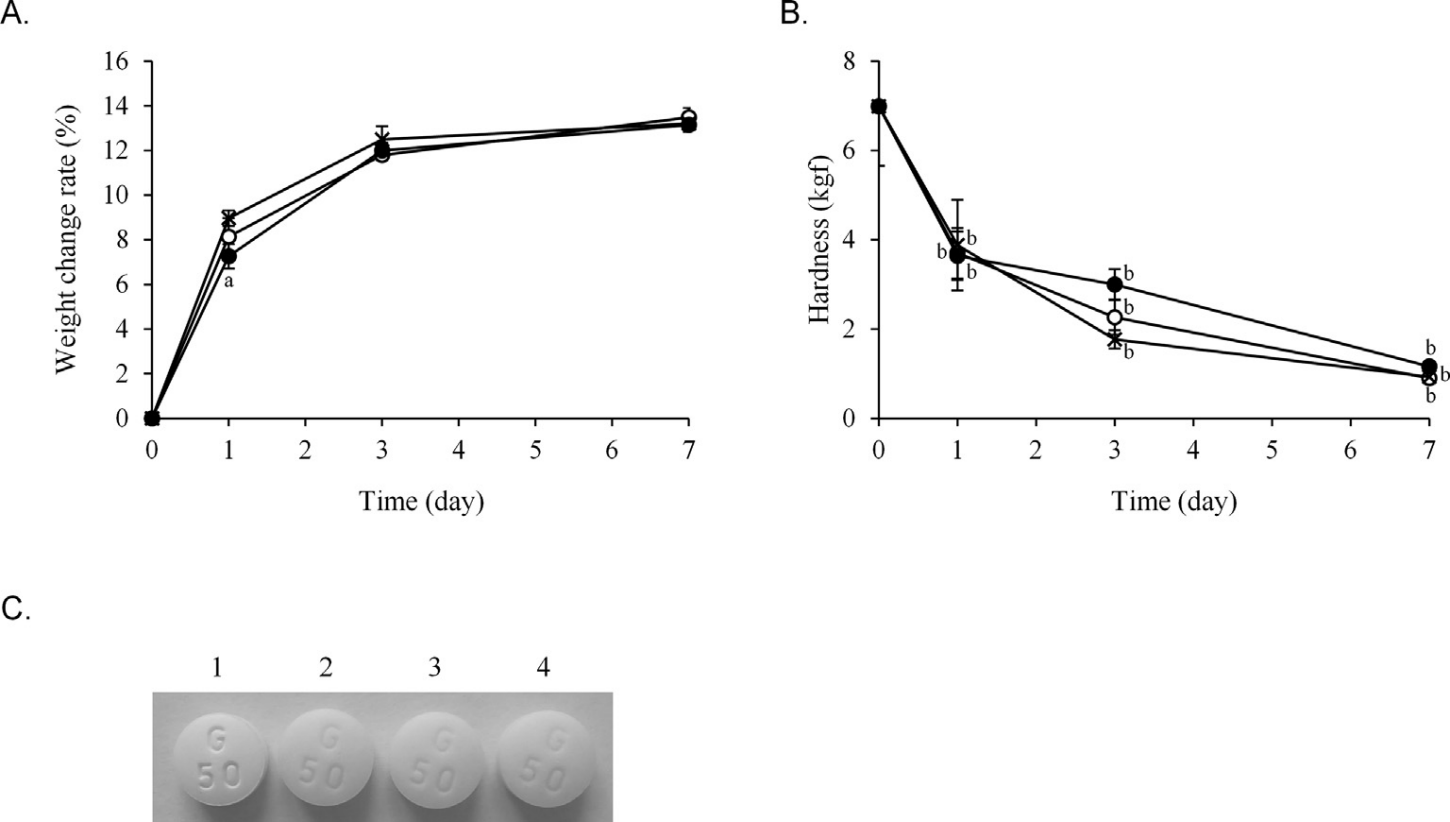
Quality change of Glucobay® tablet stored in one-dose packages. Glucobay® tablet (1 tablet) was packaged in three papers and stored for 7 days under 25 ± 1 °C and 75 ± 5% RH. Weight change (A) and hardness of tablets (B) were measured at 1, 3, and 7 days. Appearance (C) was observed at 7 days. **〇**: 20 μm thick cellophane polyethylene laminating paper, **●**: 30 μm thick cellophane polyethylene laminating paper, ×: glassine paper. Each point is mean ± SD. ^a^*P* < 0.01 (vs. glassine paper), ^b^*P* < 0.001 (vs. new tablet). 1: new tablet, 2: tablet packaged in 20 μm thick cellophane polyethylene laminating paper, 3: tablet packaged in 30 μm thick cellophane polyethylene laminating paper, 4: tablet packaged in glassine paper.

**Figure 3:**
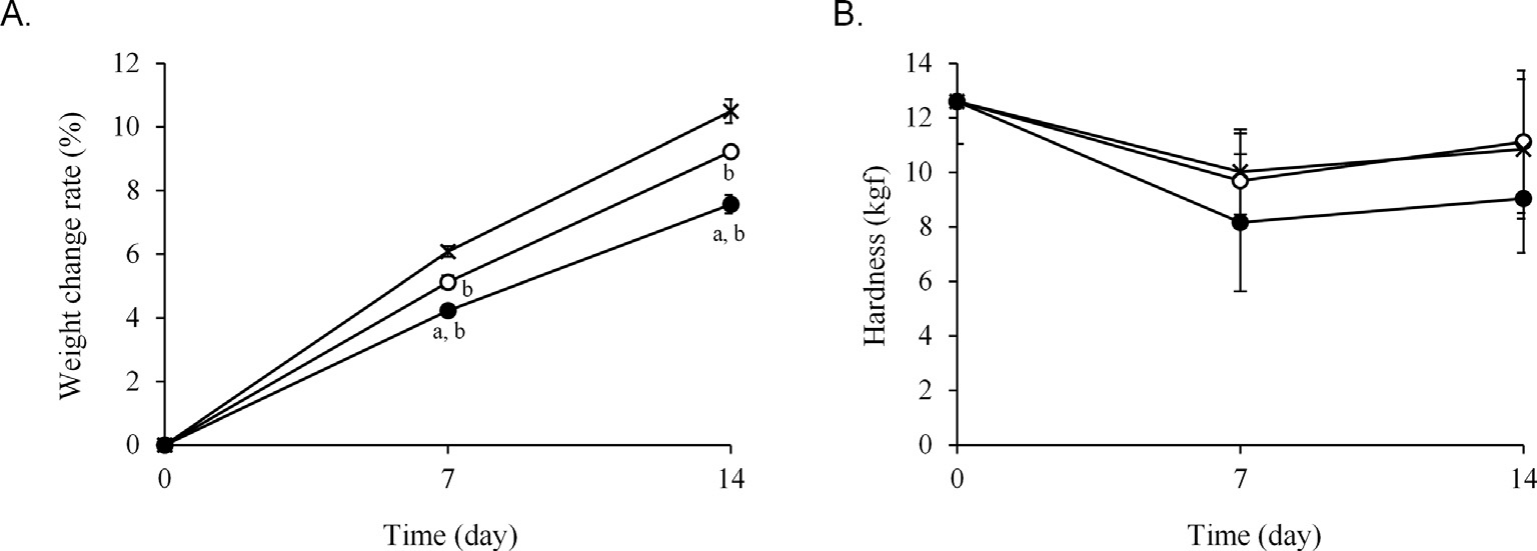
Quality change of Magmitt® tablet stored in one-dose packages. Magmitt® tablet (2 tablets) was packaged in three papers and stored for 14 days under 25 ± 1 °C and 75 ± 5% RH. Weight change (A) and hardness of tablets (B) were measured at 7 and 14 days. **〇**: 20 μm thick cellophane polyethylene laminating paper, **●**: 30 μm thick cellophane polyethylene laminating paper, ×: glassine paper. Each point is mean ± SD. ^a^*P* < 0.001 (vs. cellophane polyethylene laminating paper of thickness 20 μm), ^b^*P* < 0.001, ^b’^*P* < 0.001 (vs. glassine paper), ^c^*P* < 0.001 (vs. new tablet).

**Figure 4:**
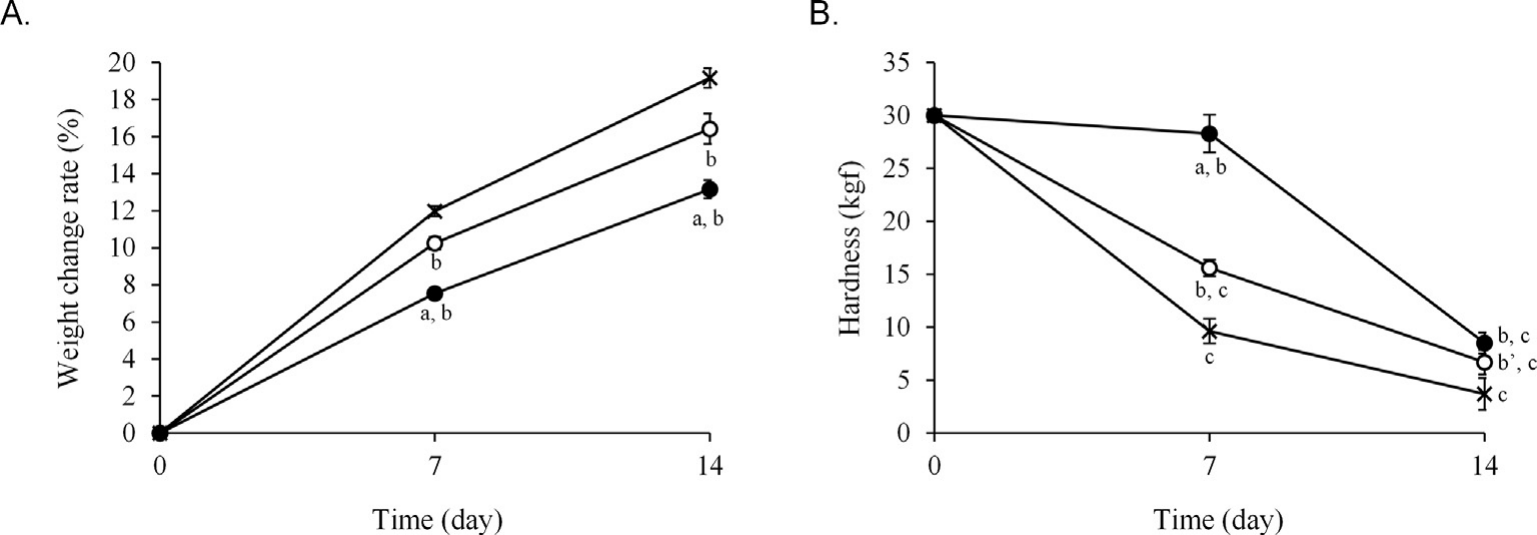
Quality change of Phosblock® tablets stored in one-dose packages. Phosblock® tablet (4 tablets) was packaged in three papers and stored for 14 days under 25 ± 1 °C and 75 ± 5% RH. Weight change (A) and hardness of tablets (B) were measured at 7 and 14 days. **〇**: 20 μm thick cellophane polyethylene laminating paper, **●**: 30 μm thick cellophane polyethylene laminating paper, ×: glassine paper. Each point is mean ± SD. ^a^*P* < 0.001 (vs. 20 μm thick cellophane polyethylene laminating paper), ^b^*P* < 0.001, ^b’^*P* < 0.01 (vs. glassine paper), ^c^*P* < 0.001 (vs. new tablet).

The weight and quality changes of the three hygroscopic medications stored in one-dose packages of each paper were examined. The weight change rate of the Glucobay® tablet packaged in 20 μm and 30 μm thick cellophane polyethylene laminating paper and glassine paper on day 1 was 8.1%, 7.3%, and 9.0%, respectively (Figure 2A). However, the difference decreased in a time-dependent manner and, after 7 days, the weight change was similar among all packaging papers. The hardness of the tablets significantly decreased from day 1 in all papers (Figure 2B). Additionally, the appearance of the tablets changed in all papers after 7 days but this change was not different among the three papers (Figure 2C). The weight of Magmitt® and Phosblock® tablets was lowest in 30 μm thick cellophane polyethylene laminating paper after 7 and 14 days (Figures 3A, 4A). The hardness of the Magmitt® tablet hardly changed (Figure 3B), whereas the hardness of the Phosblock® tablet decreased in order of glassine paper, 20 μm thick cellophane polyethylene laminating paper, and 30 μm thick cellophane polyethylene laminating paper (Figure 4B).

### 3.3 RH in one-dose packages with hygroscopic medications

**Figure 5:**
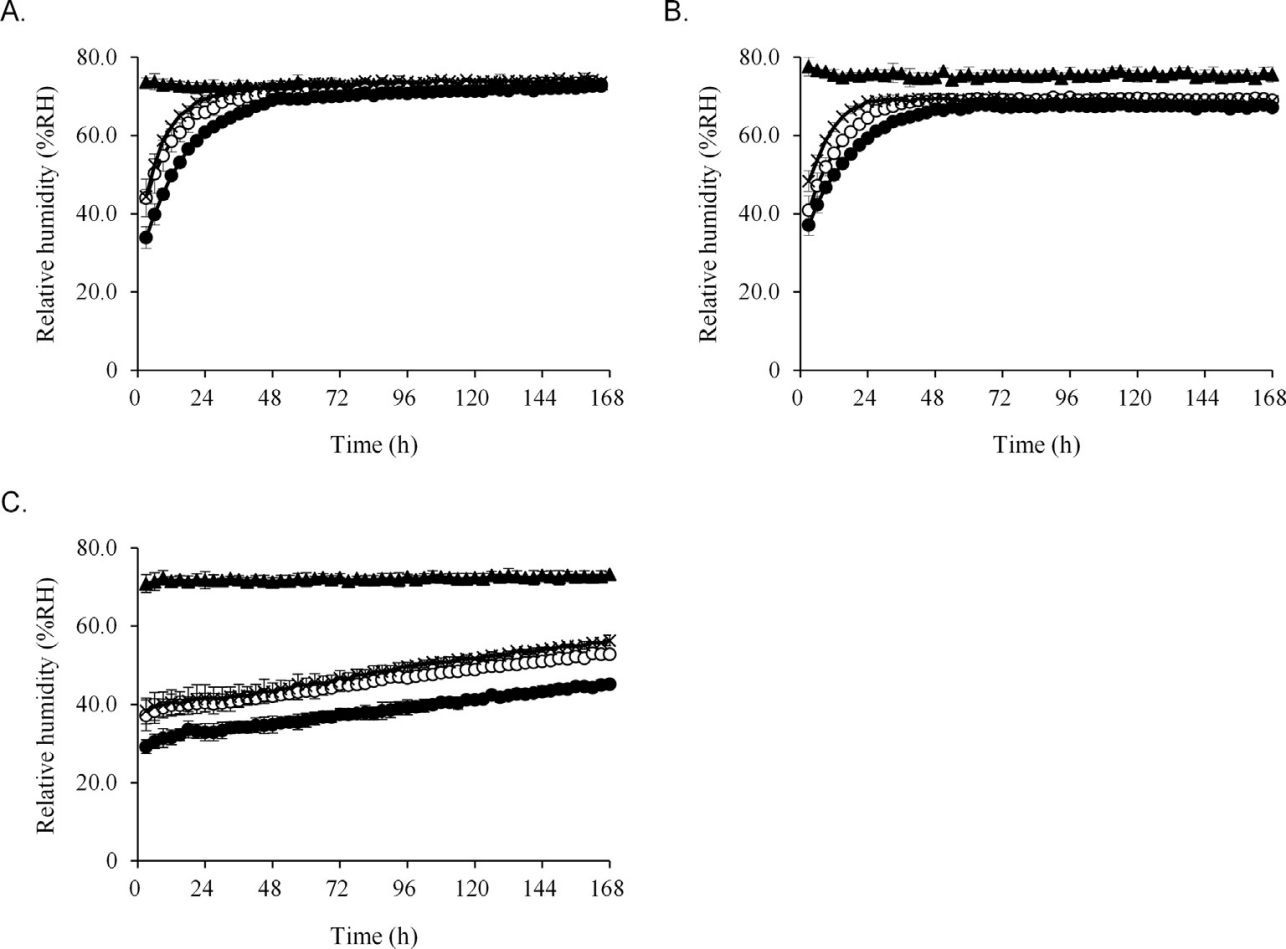
Relative humidity in packaging papers with hygroscopic tablets. The packages with Glucobay® tablet (A), Magmitt® tablet (B), and Phosblock® tablet (C) were packaged in three papers. The packaged papers were stored under 25 ± 1 °C and 75 ± 5% RH. The relative humidity in packages was measured from 3 h to 168 h. **▲**: external, **〇**: 20 μm thick cellophane polyethylene laminating paper, **●**: 30 μm thick cellophane polyethylene laminating paper, ×: glassine paper. Each point is mean ± SD.

**Figure 6:**
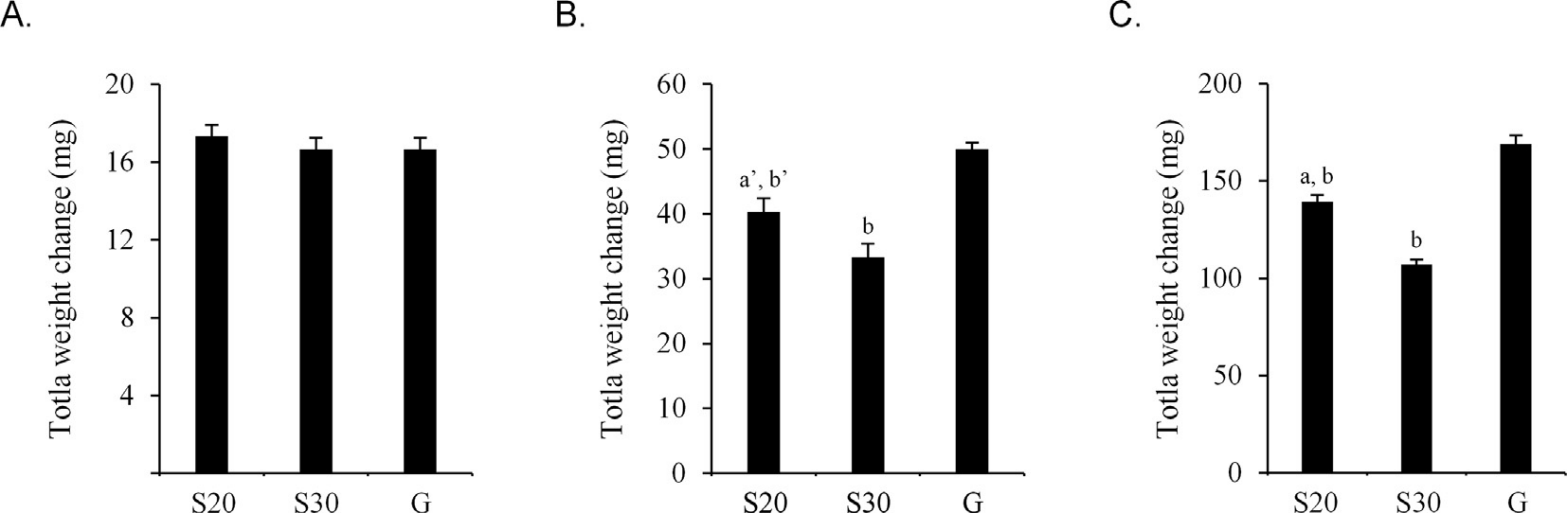
Total weight change of the tablets stored in packaging papers. Total weight change of Glucobay® tablet (A), Magmitt® tablet (B), and Phosblock® tablet (C) packaged in each paper was examined at 168 h. S20: 20 μm thick cellophane polyethylene laminating paper, S30: 30 μm thick cellophane polyethylene laminating paper, G: glassine paper. ^a^*P* < 0.001, ^a’^*P* < 0.01 (vs. 30 μm thick cellophane polyethylene laminating paper), ^b^*P* < 0.001, ^b’^*P* < 0.01 (vs. glassine paper).

We next compared the RH in one-dose packages with the medications. In packages with the Glucobay® tablet, the RH in 20 μm and 30 μm thick cellophane polyethylene laminating paper and glassine paper at 24 h was 63.8%, 60.5%, and 68.5%, respectively. These values were lower than that in the external environment, which was 70.2% (Figure 5A). However, after 168 h, the difference among these three papers was reduced and the RH was similar to that of the external environment, which was 73.7%. In the Magmitt® tablet, the RH in 20 μm and 30 μm thick cellophane polyethylene laminating paper, glassine paper, and the external environment at 24 h was 64.5%, 59.4%, 68.7%, and 75.7%,respectively (Figure 5B). After 168 h, the RH in these three papers was similar to that of the external environment, which was 75.6%. The result was similar to that in packages with the Glucobay® tablet. The RH in packages with the Phosblock® tablet in 20 μm and 30 μm thick cellophane polyethylene laminating paper, glassine paper, and external environment were 40.5%, 32.9%, 41.6%, and 71.8% at 24 h and 52.9%, 45.3%, 56.3%, and 73.3% at 168 h, respectively (Figure 5C). These differences were maintained for 168 h. The total weight changes of Glucobay®, Magmitt®, and Phosblock® tablets stored in one-dose packages after 168 h were 17.0–18.0 mg, 33.3–50.0 mg, and 107.0–169.0 mg, respectively (Figure 6).

### 3.4 Comparison of the RH in packaging papers and changes in the quality of Phosblock® tablets at 35 °C and 75% RH

**Figure 7:**
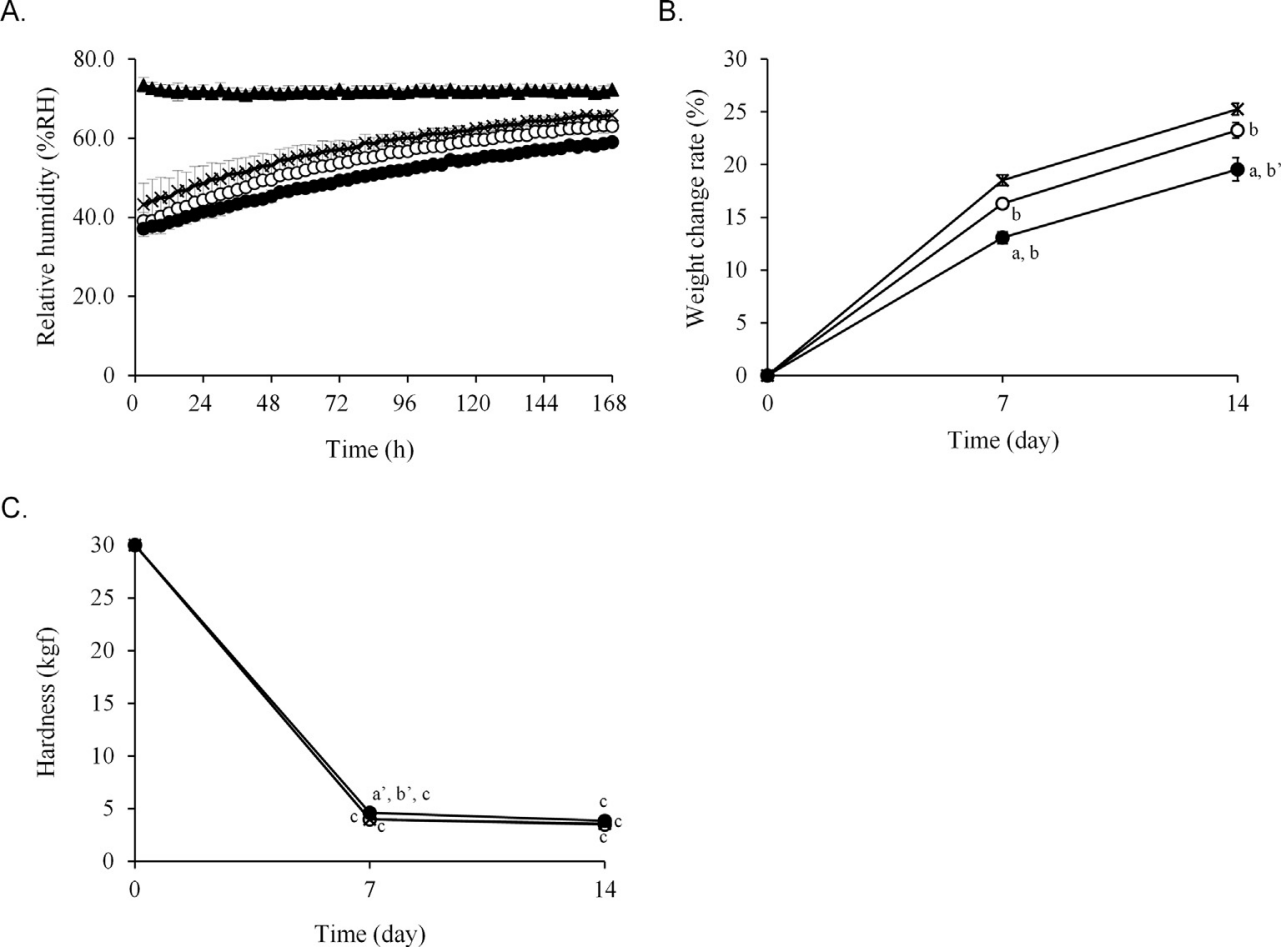
Relative humidity in packaging papers and changes in the quality of Phosblock® tablets stored under conditions of high temperature and humidity. Phosblock® tablets (4 tablets) were packaged in three types of paper and stored for 7 or 14 days at 35 ± 1 °C and 75 ± 5% relative humidity. The relative humidity in packages (A), and the weight change (B) and hardness (C) of the tablets were measured. **▲**: external, **〇**: 20 μm thick cellophane polyethylene laminating paper, **●**: 30 μm thick cellophane polyethylene laminating paper, ×: glassine paper. Each point represents the mean ± SD. ^a^*P* < 0.001, ^a’^*P* < 0.01 (vs. cellophane polyethylene laminating paper of thickness 20 μm), ^b^*P* < 0.001, ^b’^*P* < 0.01 (vs. glassine paper), ^c^*P* < 0.001 (vs. new tablet).

Measurement of the RH of the three types of packaging paper containing Phosblock® tablets stored at 35 °C and 75% RH indicated that the relationships among the RH of 20 μm and 30 μm thick cellophane polyethylene laminating papers and glassine paper were similar to those stored at 25 °C (Figure 7A). However, the RH values in all the packaging papers were higher than those recorded at 25 °C. The weight of Phosblock® tablets increased in the order of glassine paper, 20 μm thick cellophane polyethylene laminating paper, and 30 μm thick cellophane polyethylene laminating paper, which was similar to the pattern observed at 25 °C (Figure 7B). In contrast, all the weight change rates at 35 °C were higher than those at 25 °C (Figures 4A, 7B). Additionally, the hardness of the tablets stored in all types of packaging papers was significantly reduced after 7 days (Figure 7C).

## 4 Discussion

In this study, we examined the RH and quality change of hygroscopic medications in three packaging papers that are widely used in clinical settings. The RH in these packaging papers without medications was similar to that in the external environment, whereas the RH with hygroscopic medications was lower than that in the external environment. Among the papers, the RH decreased in the order of glassine paper, 20 μm thick cellophane polyethylene laminating paper, and 30 μm thick cellophane polyethylene laminating paper. In package papers without hygroscopic medications, water may have invaded the packaged papers until a RH that was similar to that of the external environment was achieved and maintained. With hygroscopic medications, the RH in packaged papers may have been determined by the balance between the invasion of water into the packaging and the moisture content of the hygroscopic medication.

The weight change and qualities of packaged hygroscopic medications were associated with the RH in each package. Thus, the moisture absorption of tablets packaged in 30 μm thick cellophane polyethylene laminating paper was the lowest. It has already been reported that the water permeabilities of cellophane polyethylene laminating paper at 20 μm and 30 μm and glassine paper are 3, 2, and 3 g/m^2^ in 24 h (20 °C, 60% RH) and 15, 11, and 16 g/m^2^ in 24 h (30 °C, 90% RH), respectively, by Yuyama, Ltd [9]. In magnesium oxide tablets, moisture absorption causes disintegration delay [6, 10, 11]. We have previously reported that the disintegration time of Magmitt® 330 mg, packaged in 20 μm thick cellophane polyethylene laminating paper and stored at 25 °C and 75% RH, is extended by approximately 51 s after 4 weeks and 9 min after 8 weeks compared with that of a non-packaged tablet (7 s) [12]. For the 2 week storage in this study, the disintegration time of the tablets was likely not a significant problem. However, as tablets in the 30 μm thick polyethylene laminating paper were less susceptible to moisture absorption, this paper might be useful for the long-term storage of tablets.

Although the moisture absorption and hardness reduction of Glucobay® and Phosblock® tablets packaged in 30 μm thick cellophane polyethylene laminating paper were suppressed the most at first, the difference among packaging papers was not detected later in the experiment. In hygroscopic medications, which absorbed moisture easily, it is difficult to maintain the medication quality for long under a high-humidity environment, even when a 30 μm thick cellophane polyethylene laminating paper was used. However, we have previously reported that the period of quality maintenance of Glucobay® tablet packaged with Magmitt® tablet in 20 μm thick cellophane polyethylene laminating paper is longer than that of the Glucobay® tablet packaged alone [5, 7]. Thus, a one-dose package of multiple hygroscopic medications may decrease the moisture absorption of each medication. In this case, a one-dose package of multiple hygroscopic medications using 30 μm thick cellophane polyethylene laminating paper may extend the period of quality maintenance compared with other one-dose packages.

Currently, a number of hygroscopic medications are inappropriately stored in one-dose packages. Thus, information on the hygroscopic medications that are suitable and problematic for one-dose packaging is necessary for clinical use. It is desirable that the preservation period of each hygroscopic medication stored in a one-dose package be established based on the association between the absorbency of each hygroscopic medication and quality reduction by moisture absorption in one-dose packaging. Additionally, we found that hygroscopic medications stored at high temperature (35 °C) absorbed moisture more readily than those stored at low temperature (25 °C), even if the medications were stored under the same RH conditions. In the case of the one-dose packaging of hygroscopic medications, it is accordingly necessary to take into consideration both the temperature and RH in different countries and seasons. For medications that readily undergo deterioration as a consequence of moisture absorption, the using of a drying agent to store the one-dose packages could make an important contribution to ensuring the safety of these products during storage [4].

In conclusion, the differences among packaging papers regarding RH, moisture absorption, and quality changes of packaged hygroscopic medications were demonstrated. We expect that the results will be used by hospitals and clinical pharmacies to understand the characteristics of packaging papers and ensure appropriate usage.

## Declarations

### Author contribution statement

T. Matsuo: Conceived and designed the experiments; Performed the experiments; Analyzed and interpreted the data; Contributed reagents, materials, analysis tools or data; Wrote the paper.

Y. Yoshida: Performed the experiments; Analyzed and interpreted the data.

T. Tomita: Conceived and designed the experiments.

Y. Sadzuka: Contributed reagents, materials, analysis tools or data.

### Funding statement

This research did not receive any specific grant from funding agencies in the public, commercial, or not-for-profit sectors.

### Declaration of interests statement

The authors declare no conflict of interest.

### Additional information

No additional information is available for this paper.
